# Teaching Public Health And Aging Using A Workshop Model

**DOI:** 10.1093/geroni/igaf122.3401

**Published:** 2025-12-31

**Authors:** Kristine Mulhorn

**Affiliations:** Drexel University, Philadelphia, Pennsylvania, United States

## Abstract

Aging populations in low- and middle-income (LMI) countries such as Cameroon face multiple adversities such as poor healthcare access, social isolation, and financial insecurity among others. To tackle these adversities necessitates an interdisciplinary methodology that incorporates varied health approaches. Thus, this paper presents insights from workshops conducted in Cameroon as part of a Fulbright program. The interdisciplinary point of view across multiple health topics was designed to facilitate collaboration among students from nursing, public health, medical laboratory science, nutrition, and mental health disciplines. The workshops provided a platform for students with hands-on experience in community health to engage in cross-disciplinary dialogue, share expertise, and develop innovative solutions tailored to the needs of older persons in Cameroon’s rural and urban landscape. One aspect of the workshops explored the implications of fluctuating demographics and public health trends in older populations, particularly through the lens of the International Classification of Disability, Functioning, and Health (ICF). Participants evaluated real-world aging-related barriers, such as limited healthcare access in remote areas, the psychological effects of social isolation, and the financial constraints. Solutions included mobile healthcare clinics to address accessibility gap for older adults in rural regions, community-based social programs to reduce loneliness and enhance mental well-being, and microfinance initiatives aimed at providing financial stability for aging individuals. The workshop model fosters interdisciplinary collaboration, critical thinking, and problem-solving skills among students and the moderating professionals. This model offers a replicable framework that can ultimately contribute to more sustainable and impactful interventions for aging populations in LMI countries.

